# Supplementary material for: Antecedents of the responsible acquisition of computers behaviour: Integrating the theory of planned behaviour with the value-belief-norm theory and the habits variable
Source: PLoS One. 2023 Jun 2;18(6):e0286022. doi: 10.1371/journal.pone.0286022 (PMC10237383; doi:10.1371/journal.pone.0286022)
Supplement: S1 Appendix — (DOCX) [file pone.0286022.s001.docx]

###### **Appendix A: Measurement items for responsible acquisition of computer behaviour**

| **Variables** |  | **Item** | **Sources** |
| --- | --- | --- | --- |
| Responsible Acquisition of Computer Behaviour | RACB1 | I have bought energy-efficient (eco-friendly) computers. | Ajzen (1991); Murugesan (2008) |
|  | RACB2 | I have referred to Electronic Product Environmental Assessment Tool (EPEAT) before making purchase decisions. |  |
|  | RACB3 | I have bought a computer after considering its negative impact on the environment at the end of its lifecycle. |  |

### Appendix B: Measurement items for Theory of Planned Behaviours factors

| **Variables** |  | **Item** | **Sources** |
| --- | --- | --- | --- |
| Attitude Towards RACB | ATRACB1 | Buying computers responsibly is a good idea. | Ajzen (1991) |
|  | ATRACB2 | Buying computers responsibly is pleasant. |  |
|  | ATRACB3 | Buying computers responsibly makes me feel satisfied. |  |
|  | ATRACB4 | I like the idea of buying computers responsibly. |  |
| Subjective Norms | SN1 | People who influence my behaviour think that I should buy computers responsibly. | Ajzen (1991) |
|  | SN2 | People who are important to me think that I should buy computers  responsibly. |  |
|  | SN3 | The Malaysian government encourages citizens to buy computers responsibly. |  |
| Perceived Behavioural Control | PBC1 | There are insufficient eco-labels to encourage me to purchase computers responsibly. | Ajzen (1991); Boldero (1995); Tonglet et al. (2004) |
|  | PBC2 | There are few environmental advertisements that encourage me to practise responsible acquisition of computers. |  |
|  | PBC3 | I perceive that it is unreasonable to pay a higher price for computers that are produced in an eco-friendly way. |  |
|  | PBC4 | I perceive that it is difficult to find outlets that sell eco-friendly computers. |  |
|  | PBC5 | Eco-friendly computers are marketed in a way that I really find incompatible with my lifestyle. For example, I prefer that computers are marketed in a way that emphasises their price, colours and style instead of their green attributes. |  |
| Responsible Acquisition of Computer Behavioural Intention | RACBI1 | I intend to buy energy-efficient (eco-friendly) computers. | Ajzen (1991); Murugesan (2008) |
|  | RACBI2 | I intend to refer to Electronic Product Environmental Assessment Tool (EPEAT) before making purchase decisions. |  |
|  | RACBI3 | I intend to buy a computer after considerating its negative impacts on the environment at the end of its lifecycle. |  |

###### Appendix C: Measurement Items for Value Belief Norms theory

| **Variables** |  | **Item** | **Sources** |
| --- | --- | --- | --- |
| Egoistic | Ego1 | Social power | Stern et al. (1999); Steg et at. (2005) |
|  | Ego2 | Wealth |  |
|  | Ego3 | Authority |  |
|  | Ego4 | Influential |  |
|  | Ego5 | Ambitious |  |
| Altruistic | Altru1 | Equality | Stern et al. (1999); Steg et al. (2005) |
|  | Altru2 | A world at peace |  |
|  | Altru3 | Social justice |  |
|  | Altru4 | Helpful |  |
| Biospheric | Bio1 | Preventing pollution | Stern et al. (1999); Steg et al. (2005) |
|  | Bio2 | Respecting the earth |  |
|  | Bio3 | Unity with nature |  |
|  | Bio4 | Protecting the environment |  |
| New Ecological Paradigm or Environmental Concern | EC1 | The so-called “ecological crisis” facing humankind has been greatly exaggerated. | Stern et al. (1999); Steg et al. (2005) |
|  | EC2 | The earth is like a spaceship with limited room and resources. |  |
|  | EC3 | If things continue on their present course, we will soon experience a major ecological catastrophe. |  |
|  | EC4 | The balance of nature is strong enough to cope with the impacts of a modern industrial nation. |  |
|  | EC5 | Mankind is severely abusing the environment. |  |
| Awareness of Consequences-ego (ACego) | AC1 | Climate change (greenhouse effects) resulting from not practising responsible acquisition will be a serious problem for me and my family. | Stern et al. (1999); Steg et al. (2005) |
|  | AC2 | The problem of toxic substances in the air and water resulting from not practising responsible acquisition will be a serious problem for me and my family. |  |
|  | AC3 | The depletion of resources due to not practising responsible acquisition will be a serious problem for me and my family. |  |
| Awareness of Consequences-altru (ACaltru) | AC4 | Climate change (greenhouse effects) resulting from not practising responsible acquisition will be a serious problem to society, country and the next generation. | Stern et al. (1999); Steg et al. (2005) |
|  | AC5 | The problem of toxic substances in the air and water, resulting from not practising responsible acquisition will be a serious problem to society, country and the next generation. |  |
|  | AC6 | The depletion of resources due to not practising responsible acquisition will be a serious problem for society, country and the next generation. |  |
| Awareness of Consequences-bio  (ACbio) | AC7 | Climate change (greenhouse effects) resulting from not practising responsible acquisition will be a serious problem for non-human species like plants and animals. | Stern et al. (1999); Steg et al. (2005) |
|  | AC8 | The problem of toxic substances in the air and water, resulting from not practising responsible acquisition will be a serious problem to non-human species like plants and animals. |  |
|  | AC9 | The depletion of resources due to not practising responsible acquisition will be a serious problem for non-human species like plants and animals. |  |
| Ascriptions of Responsibilities | AR1 | I feel jointly responsible for greenhouse effects due to not practising responsible acquisition. | Stern et al. (1999); Steg et al. (2005) |
|  | AR2 | I feel responsible for the current environmental problems due to not practising responsible acquisition. |  |
|  | AR3 | I feel responsible for the depletion of energy resources due to not practising responsible acquisition. |  |
|  | PN1 | I feel a strong personal obligation to practise responsible acquisition. | Stern et al. (1999); Steg et al. (2005) |
|  | PN2 | I am willing to put extra effort into practising responsible acquisition. |  |
|  | PN3 | I would feel guilty if I did notpractise responsible acquisition. |  |

###### Appendix D: Items measured for Habits

| **Variables** |  | **Items** | **Sources** |
| --- | --- | --- | --- |
| Habits | HA1 | Responsible purchase of computers has become a habit for me. | Venkatesh et al., (2012); Triandis (1980) |
|  | HA2 | Practising responsible acquisition does not come naturally to me. |  |
|  | HA3 | I must practise responsible acquisition. |  |
