# Supplementary material for: Antecedents of the responsible acquisition of computers behaviour: Integrating the theory of planned behaviour with the value-belief-norm theory and the habits variable
Source: PLoS One. 2023 Jun 2;18(6):e0286022. doi: 10.1371/journal.pone.0286022 (PMC10237383; doi:10.1371/journal.pone.0286022)
Supplement: S1 Dataset — (DOCX) [file pone.0286022.s002.docx]

2 2 2 4 1 1 1 4 2 2 2 2 4 1 1 1 4 4 2 2 2 4 1 1 1 3 3 2 2 2 3 1 1 1 3 3 2 1 3 4 1 1 3 5 3 2 2 1 2 1 2 3 4 4 2 2 1 5 1 1 3 4 3 2 2 1 5 8 2 4 4 3 2 1 2 2 1 1 3 5 4 2 2 1 4 1 1 3 3 3 1 1 2 2 1 1 3 4 2 1 2 3 2 13 2 1 4 3 1 1 2 4 4 1 1 3 3 1 1 2 4 1 1 1 3 3 1 1 2 4 1 1 1 3 3 3 2 1 6 8 2 5 5 4 1 2 2 4 1 1 1 3 3 1 1 2 4 1 1 1 4 3 1 1 2 4 1 1 1 4 5 1 2 2 4 1 1 1 4 3 1 1 2 2 1 1 3 3 3 1 2 2 4 5 2 1 3 3 2 2 2 4 4 3 1 4 2 1 2 3 4 1 1 1 4 2 2 2 1 5 3 2 1 4 4 3 1 2 4 1 1 6 4 4 1 1 2 2 1 1 7 3 3 1 1 2 1 1 1 1 3 3 2 2 2 3 4 3 11 3 3 4 4 4 2 4 4 4 4 4 4 3 4 4 4 3 3 3 3 3 3 3 3 3 3 4 4 4 4 3 3 3 3 3 3 3 3 5 5 5 3 5 5 4 4 4 4 4 4 4 4 4 4 4 4 3 3 5 2 3 4 4 4 4 4 4 5 2 5 5 4 5 4 4 4 5 4 4 4 3 3 3 3 3 4 4 4 4 4 4 4 3 4 5 2 3 4 4 4 4 3 3 4 2 2 2 4 4 3 3 3 3 3 3 3 3 3 3 3 3 3 3 3 3 4 4 4 3 3 3 3 3 3 1 1 1 4 4 4 4 4 4 4 4 4 3 3 3 3 4 4 3 3 3 3 4 5 3 4 4 3 3 3 4 4 4 5 4 3 5 5 5 3 4 4 2 4 3 2 2 2 2 3 3 3 2 3 3 3 3 3 3 4 2 3 3 2 2 2 4 4 5 1 4 4 3 3 3 2 3 4 2 2 4 3 3 3 4 4 4 4 4 4 4 4 4 4 4 5 4 4 4 4 5 4 3 3 3 3 3 3 3 3 3 3 3 4 2 2 2 3 3 4 3 3 4 3 5 4 4 4 4 4 4 4 4 4 4 4 4 4 2 2 3 3 3 3 2 2 3 4 4 4 3 3 3 4 4 4 2 3 3 3 3 3 3 3 3 4 4 4 5 5 5 5 5 5 3 3 3 3 3 3 4 4 4 3 3 3 3 3 3 3 3 3 5 4 4 4 4 4 4 4 4 5 5 5 4 4 4 3 3 3 2 2 2 4 4 4 4 4 4 3 3 3 4 4 4 4 4 4 3 3 2 3 3 3 2 2 2 3 3 3 3 3 3 3 3 3 3 3 3 4 4 4 3 3 3 2 2 2 1 1 1 3 3 3 5 4 5 5 4 4 4 4 4 3 3 3 4 4 4 4 4 4 4 4 3 3 3 3 4 4 4 5 5 5 2 2 2 4 4 4 2 2 2 1 1 1 3 3 3 3 3 3 2 2 2 3 3 2 2 2 2 3 3 3 3 3 3 2 3 2 3 3 3 3 3 3 3 3 3 3 3 3 3 3 3 4 4 4 4 4 4 4 4 4 5 5 4 3 3 3 4 4 4 3 3 3 3 3 3 3 3 3 3 3 3 2 2 2 3 3 3 4 4 4 3 3 3 3 3 3 4 4 4 4 4 4 4 4 4 3 3 3 3 3 3 3 3 3 3 3 3 3 3 3 3 3 3 3 3 3 3 3 3 3 2 2 4 4 4 3 5 5 4 5 5 4 4 4 4 4 4 4 3 3 4 4 3 3 4 4 4 4 4 4 4 4 4 4 4 5 4 4 3 3 3 3 3 3 3 3 3 4 4 4 4 2 2 2 4 4 4 4 4 4 4 4 4 4 4 3 3 3 3 3 3 3 3 3 3 3 3 3 3 3 3 3 3 3 3 3 3 3 3 3 3 3 3 3 3 3 4 4 4 4 4 4 4 4 4 5 5 5 4 4 4 4 4 4 4 4 4 3 3 3 3 3 3 3 4 3 4 4 3 3 3 3 4 4 4 1 1 3 3 3 3 2 2 2 3 3 2 3 3 2 2 2 2 3 3 3 3 3 3 2 2 2 4 3 3 3 3 3 3 3 3 2 2 3 3 3 3 3 3 3 3 3 4 4 4 4 4 4 4 4 4 4 4 4 4 4 4 4 4 4 3 3 3 3 3 3 3 3 3 3 3 3 3 3 3 3 3 3 4 4 4 4 4 4 4 4 4 4 4 4 4 4 4 4 4 4 3 3 3 3 3 3 3 3 3 3 3 3 3 3 3 3 3 3 2 3 3 3 3 3 3 3 3 5 4 4 4 4 4 4 4 5 3 4 4 4 4 4 4 4 3 4 3 3 3 3 3 3 3 4 5 4 4 4 4 5 5 4 4 3 4 4 4 4 4 4 4 4 4 4 3 4 4 3 4 4 4 3 4 4 4 4 3 3 3 4 3 3 3 3 3 3 3 3 3 3 3 3 3 3 3 3 3 3 3 3 3 3 3 3 3 3 3 4 1 1 1 1 2 2 2 2 4 5 5 5 5 4 4 4 4 3 3 3 3 3 4 4 4 3 4 4 4 4 4 3 3 3 3 1 1 1 1 1 4 4 4 4 3 2 2 2 2 1 1 1 4 3 3 3 3 3 2 2 2 3 3 4 3 3 3 4 4 4 2 2 2 2 2 2 3 3 3 4 3 3 3 3 3 3 3 3 3 4 4 4 4 4 4 4 4 4 4 4 4 4 4 4 4 4 4 3 3 3 3 3 3 3 3 3 3 3 3 3 3 2 3 2 3 4 2 2 2 2 3 3 3 4 4 4 4 4 4 4 4 4 2 3 3 3 3 3 3 3 3 4 3 3 3 3 3 3 3 3 2 3 3 3 3 3 3 3 3 2 5 5 5 5 5 5 5 5 1 3 3 3 3 3 3 3 3 2 4 4 4 4 4 4 4 4 2 4 4 4 4 4 4 4 4 2 4 4 4 4 4 4 4 4 2 4 4 4 4 3 4 4 3 1 4 4 4 4 4 4 4 4 1 3 2 3 3 2 2 3 3 1 3 3 3 3 3 3 3 3 2 3 3 3 3 3 3 3 3 2 2 2 2 2 2 2 2 2 2 4 4 4 4 4 4 4 4 2 3 3 3 3 3 3 3 3 2 3 3 3 3 3 3 3 3 2 4 4 4 4 4 4 4 4 5 4 3 3 3 3 3 3 3 4 3 3 4 4 4 4 4 4 3 2 2 3 3 3 3 3 3 1 4 4 4 4 4 4 4 4 3 3 3 3 3 3 3 3 3 1 4 4 4 4 4 4 4 4 1 4 4 4 4 4 4 4 4 2 3 3 3 3 3 3 3 3 2 3 3 3 3 3 3 3 3 4 4 4 4 4 4 4 4 4 3 2 2 2 2 4 4 4 4 3 4 3 4 4 3 4 3 4 3 1 2 2 2 4 4 4 4 5 2 2 2 2 4 4 4 4 4 1 2 1 1 4 4 4 4 3 2 2 2 2 4 4 4 4 2 2 2 2 2 3 3 3 3 3 2 2 2 2 5 5 5 5 3 2 3 3 2 4 4 4 4 3 1 2 2 1 4 4 4 4 2 1 1 1 1 4 4 4 4 3 1 1 1 1 2 2 2 2 1 2 2 2 2 3 3 3 3 2 2 1 2 2 3 3 3 3 5 2 1 1 1 3 3 3 3 3 2 2 2 2 4 4 4 4 3 2 2 3 2 3 3 3 3 2 2 3 3 2 3 3 3 3 5 5 4 4 4 3 3 3 3 3 4 4 4 4 2 2 2 2 2 3 3 3 3 4 4 3 4 3 2 2 2 2 2 2 2 2 5 3 3 3 3 2 2 2 2 5 2 2 2 2 3 3 3 3 4 2 1 2 2 4 4 4 4 3 2 1 2 2 4 4 4 4 3 3 3 3 3 3 3 3 3 4 4 4 4 4 4 4 4 4 3 3 3 3 3 3 3 3 3 4 3 3 3 4 4 4 4 4 3 3 3 2 2 1 3 2 5 4 4 4 4 4 4 4 4 4 4 5 5 4 4 4 3 3 3 5 5 4 4 4 1 1 2 4 4 4 4 4 3 3 3 5 5 5 5 5 3 3 3 4 4 5 4 4 2 2 2 5 4 4 4 4 2 2 3 5 5 5 5 5 3 3 3 4 4 4 4 5 2 2 2 3 3 3 3 3 2 1 1 5 5 5 5 5 5 5 5 4 4 4 4 4 3 3 3 4 4 4 4 4 3 2 3 5 4 4 4 4 1 2 2 4 4 3 3 3 5 5 5 4 3 4 4 4 3 3 3 2 2 2 2 1 1 2 2 1 1 1 1 2 3 3 3 5 4 4 5 5 5 4 5 3 3 3 3 3 5 5 5 3 3 3 3 3 4 4 4 2 3 3 3 3 3 3 3 4 4 4 4 4 3 3 3 5 4 4 4 4 4 4 4 2 4 4 4 4
