# Supplementary material for: Antecedents of the responsible acquisition of computers behaviour: Integrating the theory of planned behaviour with the value-belief-norm theory and the habits variable
Source: PLoS One. 2023 Jun 2;18(6):e0286022. doi: 10.1371/journal.pone.0286022 (PMC10237383; doi:10.1371/journal.pone.0286022)
Supplement: Attachment — Submitted filename: Plos one_reviewers comments.docx [file pone.0286022.s003.docx]

This study focuses on discovering the antecedents of Responsible Acquisition of Computer Behaviour (RACB) which is a form of RCB. The study used TPB, VBN and habits variable to explain RACB. I have the following comments:

- More updated literature is required in 2.3 Perceived Behavioural Control and 2.5 Values and New Ecological Paradigm 2.6 Awareness of Consequences and 2.9 Habits. Please read the following papers:
  - Wut, T. M., & Ng, P. M. L. (2022). Perceived CSR motives, perceived CSR authenticity, and pro-environmental behavior intention: an internal stakeholder perspective. *Social Responsibility Journal*, (ahead-of-print).
  - Ng, P. M. L., & Cheung, C. T. Y. (2022). Why do young people do things for the environment? The effect of perceived values on pro-environmental behaviour. *Young Consumers*, (ahead-of-print).
  - Yuriev, A., Dahmen, M., Paillé, P., Boiral, O., & Guillaumie, L. (2020). Pro-environmental behaviors through the lens of the theory of planned behavior: A scoping review. *Resources, Conservation and Recycling*, *155*, 104660.
- Is habit a theory? You mentioned in the abstract that “This research aimed to investigate the antecedents of responsible acquisition of computers behaviour (RACB) among Malaysian consumers by using an integrated model of the three theories”. Please revise accordingly.
- The following hypotheses are missing but the arrows have been shown in Figure 1:
  - Attitude 🡪 Responsible acquisition of green computers behavioural intention
  - SN 🡪 Responsible acquisition of green computers behavioural intention
  - PBC 🡪 Responsible acquisition of green computers behavioural intention
- Why 5-Likert scale is adopted? Any empirical support?
- Appropriate pre-test and pilot study
- Figure 1 and Figure 2 are difficult to follow. Please make a similar approach (especially H11, habit)
- For the implications, please divide them into (1) theoretical and (2) practical implications.
- Literature support in implications is insufficient. Please add more updated literature accordingly.
