# Supplementary material for: Antecedents of the responsible acquisition of computers behaviour: Integrating the theory of planned behaviour with the value-belief-norm theory and the habits variable
Source: PLoS One. 2023 Jun 2;18(6):e0286022. doi: 10.1371/journal.pone.0286022 (PMC10237383; doi:10.1371/journal.pone.0286022)
Supplement: Attachment — Submitted filename: PLOS One Comment and Response.docx [file pone.0286022.s004.docx]

Reviewer #1:

Comment:

More updated literature is required in 2.3 Perceived Behavioural Control and 2.5 Values and New Ecological Paradigm 2.6 Awareness of Consequences and 2.9 Habits. Please read the following papers:
o Wut, T. M., & Ng, P. M. L. (2022). Perceived CSR motives, perceived CSR authenticity, and pro-environmental behavior intention: an internal stakeholder perspective. Social Responsibility Journal, (ahead-of-print).
o Ng, P. M. L., & Cheung, C. T. Y. (2022). Why do young people do things for the environment? The effect of perceived values on pro-environmental behaviour. Young Consumers, 23, 539-554.
o Yuriev, A., Dahmen, M., Paillé, P., Boiral, O., & Guillaumie, L. (2020). Pro-environmental behaviors through the lens of the theory of planned behavior: A scoping review. Resources, Conservation and Recycling, 155, 104660.

Response:

We have updated the literature for Section 2.3, 2.5, 2.6, and 2.9, and included the following 8 most recent journal articles as our references.

1. Ates, H. (2020). Merging theory of planned behaviour and value identity personal norm model to explain pro-environmental behaviour. *Sustainable Production and Consumption, 24*, 169-180.
2. Ates, H. (2021). Understanding students’ and science educators’ eco-labeled food purchase behaviours: Extension of Theory of Planned Behaviour with Self-identity, Personal Norm, Willingness to pay, eco-label knowledge. *Ecology of Food and Nutrition, 1*,1-16.
3. Ng, P. M. L., & Cheung, C. T. Y. (2022). Why do young people do things for the environment? The effect of perceived values on pro-environmental behaviour. Young Consumers, 23, 539-554.
4. Nguyen, T. N., Lobo, A. and Greenland, S. (2016). Pro-environmetnal purchase behaviour: The role of consumers’ biospheric values. *Journal of Retailing and Consumer Services. 33*, 98-108.
5. Russell, S. V., Young, C. W., Unsworth, K. L., Robinson, C. (2017). Bringing habits and emotions into food waste behaviour. *Resource Conservative Recycle, 125*, 107–114.
6. Taufique, K. M. R., & Vaithianathan, S. (2018). A fresh look at understanding green consumer behaviour among young urban Indian consumers through the lens of theory of planned behaviour. *Journal of Cleaner Production, 183,* 46-55.
7. Wut, T. M., & Ng, P. M. L. (2022). Perceived CSR motives, perceived CSR authenticity, and pro-environmental behavior intention: an internal stakeholder perspective. Social Responsibility Journal, (ahead-of-print).
8. Yuriev, A., Dahmen, M., Paillé, P., Boiral, O., & Guillaumie, L. (2020). Pro-environmental behaviors through the lens of the theory of planned behavior: A scoping review. Resources, Conservation and Recycling, 155, 104660.

Comment:

• Is habit a theory? You mentioned in the abstract that “This research aimed to investigate the antecedents of responsible acquisition of computers behaviour (RACB) among Malaysian consumers by using an integrated model of the three theories”. Please revise accordingly.

Response:

The sentences in the abstract has been revised to “This research aimed to investigate the antecedents of responsible acquisition of computers behaviour (RACB) among Malaysian consumers integrating the TPB, VBN, and Habits variable.”

Comment:
• The following hypotheses are missing but the arrows have been shown in Figure 1:
o Attitude ◊ Responsible acquisition of green computers behavioural intention
o SN ◊ Responsible acquisition of green comptuters behavioural intention
o PBC ◊ Responsible acquisition of green computers behavioural intention

Response:

In response to the comments above, the following literature reviews and hypotheses are added to the revised manuscript:

Previous research validated and substantiated that positive attitudes will lead to greater behavioural intention positively in the context of RCB, e.g. Harland et al. (1999) – pro-environmental behaviour such as using unbleached paper, energy-saving light bulbs, turning off the faucet; Follow & Jobber (2000) – environmentally purchasing behaviour; Chan & Lau (2008) – green-purchase behavior; Nguyen et al., (2016)- purchase behaviour of energy efficient household appliances; Joshi & Rahman (2017) – sustainable purchasing behaviour; Ates, (2021) – eco-labeled food purchase. Thus, it is expected that Malaysian consumers who have positive attitude towards RACB will be more likely to engage themselves in RACB. Henceforth, the following hypothesis is postulated.

H1: Attitude towards RACB positively affect an individual’s intention of engaging RACB.

SN is found have direct and significant effects on behavioural intentions to perform responsible purchasing behaviour (e.g. Dean et al., 2012; Paul et al., 2015; Muhammad et al., 2019). The strength of SN is relied on “normative belief” and “motivation to comply”. “Normative belief” refers to whether others think the individual should or should not perform responsible purchasing behaviour, whereas “motivation to comply” refers to the individual’s motivation to comply with society (Paul et al., 2015). Similarly, this study demonstrates that the consumers’ referent groups such as family members, friends or teachers’ opinions and expectations will influence their intention of practising RACB. As such, the following hypothesis is formed.

H2a: Subjective Norms positively affect an individual’s intention of engaging RACB.

In line with the TPB, the PBC predicts specific responsible purchasing behaviour directly and indirectly through intentions (Azjen,1999). Prior studies supported the findings where PBC influenced pro-environmental responsible purchasing behavioural intention and actual responsible purchasing behaviour, ranging from recycling to green purchasing of household appliances (McCarty & Shrum, 2001). Researchers like Ates (2020); Yuriev et al (2020) delineated that consumers with high control over the barriers or discomfort in pro-environmental behaviour will likely involve themselves in pro-environmental behaviour. In a similar vein, Malaysian consumers who perceive that there are conducive external conditions like the availability of eco-label, advertisements related to RACB, green computers, and so forth will spur their RACB either directly or indirectly via behavioural intention. Therefore, the following hypotheses were formed.

H3a: Perceived behavioural control positively affects an individual’s intention of engaging RACB.

Comment:
• Why 5-Likert scale is adopted? Any empirical support?

Response:

The following explanation is added to the revised manuscript:

The items measuring the TPB and VBN variables were assessed on a five-point Likert scale, which have been validated in previous studies (Sawitri et al., 2015; Fu et al., 2021). Revilla et al. (2014)’s findings evidenced that a five-point scale is the best scaling technique for agree and disagree questions in terms of data quality and response rate.

References that support the use of 5-Likert scale are also added to the revised manuscript:

1. Sawitri, D. R., Hadiyanto, H. and Hadi, S. P. (2015). Pro-environmental behaviour from a Social Cognitive Theory Perspective. *Procedia Environmental Sciences.* 23, 27-33.
2. Fu, W., Zhou, Y., Li, L. and Yang, R. (2021). Understanding household electricity-saving behaviour: Exploring the effects of perception and cognition factors. *Sustainable Production and Consumption, 28*, 116-128.
3. Revilla, M. A., Saris, W. M. and Krosnick, J. A. (2014). Choosing the number of categories in Agree-Disagree scales. *Sociological Methods & Research, 43*, 73-97.

Comment
• Figure 1 and Figure 2 are difficult to follow. Please make a similar approach (especially H11, habit)

Response:

Both figures have been re-drawn. Figure 1 reflects the combination of TPB, and VBN models together with one additional variable (habits). We’ve included the hypotheses Attitude->RACBI, SN-> RACBI, PBC->RACBI and RACBI->RACB accordingly. Changes have been made for Figure 2 as well.

Comment
• For the implications, please divide them into (1) theoretical and (2) practical implications.

Response:

The following theoretical and practical implications have been discussed in section 5.4 and 5.5.

5.4 Theoretical Implications

The literature in responsible purchasing behaviour studies is enriched since there is an absence of an empirical study that examines the level of RCB and the antecedents of adopting RCB with respect to computer acquisition (high-cost purchase decision) by individual consumers, particularly in emerging economies, with fast-growing computer penetration (Worldometers, n.d) in a collectivist culture comprise low-income and middle-income groups.

The analysis of this study provides holistic views relating to integrating the TPB, and VBN models with habit variables in the high-cost pro-environmental behaviour domain. Thus, filling the literature gaps where most of the scholars either dived into individual models like TPB (e.g., Lago, 2020; Kang & Moreno, 2020) or VBN (e.g. Sharma and Gupta, 2020), or extended individual models with additional variables (e.g. Ates, 2021). The present findings validated that the cognitive deliberative process occurs, and self-interest variables are significant wherein the environmental behaviour is costly. Also, biospheric value is the main factor that triggered the TPB (self-interest aspect) and VBN (morality aspect) variables in forming decisions to adopting RACB. This further re-affirmed the biospheric value is distinct from altruistic value in the context of pro-environmental behaviour (Nguyen, 2016; Ates, 2021). SN is direct predictor of RACB (TPB variable), PN (VBN variable), AR (VBN variable) and HA (TIB). This further substantiated that SN is a very important element in the context of developing countries with collectivist cultures. Besides, HA are found to affect intention but not RACB directly. This implies that in high-cost pro-environmental behaviour, individuals’ decisions will be affected by their HA through their reasoning and assessing of the costs and benefits of each alternative (i.e. RACBI).

5.5 Practical Implications

Governments and environmental NGOs can make biospheric values more salient and instill them through informational strategies in formal and informal education, which will ultimately influence individuals’ pro-environmental beliefs, intentions and behaviour (Nguyen et al., 2019; Dharmesti et al., 2020). For formal education, core subjects/syllabuses offered in elementary, secondary and tertiary education could incorporate environmental issues related to purchase decisions. For informal environmental education, seminars, exhibitions, radio shows, drawing or colouring competitions, workshops or the mass media such as television, newspapers, the Internet or during social interaction could be used to create public awareness about the importance of biospheric values (Hassan & Pudin, 2011; Dharmesti et al., 2020**).**

Manufacturers can assist consumers to be informed that their purchasing decisions are in line with biospheric values through voluntary labelling. According to the Organisation for Economic Co-operation and Development (OECD) 2008 report, multi-criteria labels, which compare products with others in the same category in terms of environmental impact throughout their life cycles, as well as single-issue labels (e.g. the Energy Star label) which specifies environmental issue addressed by the product (e.g. energy efficiency), are most useful in communicating complex information about a particular product, thus, enabling customers to make informed choices. Moreover, consumers perceive that purchasing eco-label products is an environmentally friendly behaviour compare to conventional products (Ates, 2021). In this regards, marketers should be informed of the environmental benefits contributed by Energy Star-label computers. Hence, such information could be delivered to the final consumers to enhance their understanding of the importance of practising RACB to protect the environment, and make them aware such behaviour is congruent with biospheric values.

Apart from strengthening the biospheric values, the extended TPB also indicates the relationships between SN and HA will elicit RACBI. Additionally, the SN is found to have direct impact on RACB and the two VBN variables, i.e. PN and AR. The government could build a strong social support for RACB among consumers, which could affect others’ habits, perceptions of their responsibility for their actions on the environment, their personal moral obligation in practising RACB and their behaviour, via policy-making and social marketing. Government could limit the selling of cloned PCs and provide subsidies to those who purchase green computers. Nguyen (2016) and Zhang et al. (2017) suggested advertisements in the mass media, and information campaigns or education could educate the public to ensure that pro-environmental behaviour is morally and socially acceptable. This will motivate others (e.g. friends, family, government and people around the individuals) to support RACB. Subsequently, building strong social norms, which will lead to a social change of HA, PN, AR and PN.

Lin et al (2022) suggested that governments, retailers or organisations could leverage mobile applications to promote sustainable responsible consumption. Likewise, Wide Fund for Nature (WWF)--Malaysia and Microsoft Malaysia smartphone application (app) on “Earth Hour Malaysia” was a success (Microsoft, 2012). Perhaps, the government could allocate funds to WWF-Malaysia or Microsoft Malaysia or other relevant organizations to duplicate similar apps to promote and educate Malaysians about RACB. These apps could reinforce social messages with regard to the social expectations on performing RACB for the collectivist benefits, such as RACB could reduce harmful environmental impact to the next generation and society, thus providing strong social support and in turn affecting consumers’ AR and PN beliefs, habits and actual behaviour.

Comment
• Literature support in implications is insufficient. Please add more updated literature accordingly.

Response: We have updated the literature by adding the recent references as listed below.

1. Ates, H. (2021). Understanding students’ and science educators’ eco-labeled food purchase behaviours: Extension of Theory of Planned Behaviour with Self-identity, Personal Norm, Willingness to pay, eco-label knowledge. *Ecology of Food and Nutrition, 1,* 1-16.
2. Dharmesti, M., Merrilees, B., & Winata, L. (2020). “I’m mindfully green”: Examining the determinants of guest pro-environmental behaviors (PEB) in hotels. *Journal of Hospitality Marketing & Management, 1*, 1–18.
3. Lin, Y.T., Tseng, T. H., Chang, A., and Yang, C.C. (2022). A value adoption approach to sustainable consumption in retail stores. *International Journal of Retail & Distribution Management, 50*, 1412-1434.
4. Nguyen, T. N., Lobo, A. and Greenland, S. (2016). Pro-environmetnal purchase behaviour: The role of consumers’ biospheric values. *Journal of Retailing and Consumer Services. 33*, 98-108.
5. Zhang, X.J., Geng, G.J. and Sun, P (2017). Determinants and implications of citizens’ environmental complaint in China: Integrating theory of planned behaviour and norm activation model. *Journal of Cleaner Production, 166*, 148-156

Comment:

1. Hiring a professional proof-reader (native speaker) to further enhance the readability of the article, e.g.,
a. Abstract: “The research question is how to encourage such behaviour”
b. Intro: “Their studies revealed that incorporating moral/personal norms into rational model (i.e. TPB) DO account”
c. Intro: “Moreover, most of the prior RCB combined TPB and VBN studies AT research mainly focused”
d. 2.6: “This study posits that the Malaysian consumer’s environmental concern will determine”
e. “low-cost pro-environmental behaviour, its effect on high-cost behaviour remains unknown.”
->
Add “pro-environmental” after “high-cost”
f. 6. “and make them AWARE SUCH behaviour is congruent with Biospheric values.”
g. Please note that the above list is by no means exhaustive, please check for other typos/ errors

Response:

A professional proofreader is hired to proofread this manuscript to address these proofreading issues. Therefore, comments from number 1a to number 1g have been addressed accordingly.

2. Intro and 2.1: “e.g. Ates (2020) – pro-environmental behaviour; Li et al. (2021) – purchase of organic food; Bamberg and Moser (2007) – pro-environmental behaviour; and Wall et al. (2007)’s studies – travel mode choices.”
->
Please elaborate a bit more instead of just stating the keywords e.g., “pro-environmental behaviour”

Response:

The following explanations were added to the manuscript:

Responsible consumption behaviour (RCB) is a pro-environmental behaviour and was defined as the “acquisition, consumption and disposition of goods, services, time and ideas by decision-making units without harming the environment or society” (Stancu, 2011). Thogersen (1999) highlighted that it is worth studying RCB that significantly affects environmental quality, particularly at the acquisition stage, as the right responsible purchasing decision could have the potential to reduce and eliminate environmental harm in the later stages of the consumption cycle. Joshi and Rahman (2015) supported this claim by highlighting that 40% of environmental harm is caused by irresponsible consumption through the purchase of non-sustainable products.

RACB is defined as making a computer purchase without harming the environment such as buying Electronic Product Environmental Assessment Tools (EPEAT)-compliant computers. EPEAT is a standard certified by Green Electronics Council for computer product that contains less or no toxic content (e.g. heavy metals), consumes less energy, and is recyclable and easily upgradeable to extend its lifespan (Greenelectronicscouncil.org, 2018).

Comment:

3. 2.1 – 2.4: “Therefore, this study does not need hypothesis testing on the relationship” and other parts with similar meaning e.g., “The relationship between SN and behavioural intention will not need to be tested in the current study as their relationship presented in the TPB model has already been validated across various contexts.”; “The aforementioned relationships as shown in the TPB model have empirical support, thus this study will not test their relationships.”; & “This study does not test the causal relationship between intention and actual behaviour since such relationships have been validated across different context.”
->
Despite the fact that previous studies have demonstrated positive findings, it may be advisable to test the hypotheses yourself once again.

Response:

Relationships between TPB variables were included in the hypotheses (refer to H1 to H4 below) and tested in this study.

H1: Attitude towards RACB positively affect an individual’s intention of engaging RACB.

H2a: Subjective Norms positively affect an individual’s intention of engaging RACB.

H2b: Subjective Norms positively affect an individual’s engagement in RACB.

H2c: Subjective Norms positively affect an individual’s Personal Norms in RACB.

H2d: Subjective Norms positively affect an individual’s Ascription of Responsibility in RACB.

H2e: Subjective Norms positively affect an individual’s Habits in practising RACB.

H3a: Perceived behavioural control positively affects an individual’s intention of engaging RACB.

H3b: Perceived behavioural control positively affects an individual’s engagement in RACB.

H4: Behavioural intention positively affects an individual’s engagement in RACB.

Comment:

4. 2.7: “…and the causal relationships between AC beliefs and AR beliefs were not empirically tested. Hence, the following hypotheses are tested in this study”
->
Can causality claims be made?
<https://ftp.cs.ucla.edu/pub/stat_ser/r393-reprint.pdf>

Response:

The following explanations were added to the manuscript:

Stern et al. (1993) stated that awareness of adverse consequences (AC) will lead to AR. Individuals with high AC are presumed to become aware of the extensive and specific consequences of possible actions, and to adopt the perspective of those who will be affected when weighing decisions. Since AC beliefs are positively related to AR beliefs, i.e. individuals who are more aware of the adverse consequences of their action will feel more responsible to reduce the threats resulting from their action (Steg et al., 2005; Sahin, 2013), the following hypotheses are tested in this study:

*H8a: An individual’s Awareness of Consequences to self positively affects his or her Ascription of Responsibility for acquiring computers responsibly.*

*H8b: An individual’s Awareness of Consequences to others positively affects his or her Ascription of Responsibility for acquiring computers responsibly.*

*H8c: An individual’s Awareness of Consequences to the biosphere positively affects his or her Ascription of Responsibility for acquiring computers responsibly.*

Comment:
5. Were the questionnaire items in English? Did the respondents fully understand the questions?

Response:

Yes. The questionnaire items are in English. The respondents can understand the question since Malaysia adopts a bilingual system of education. Notably, English language is a compulsory subject at primary, secondary and tertiary education levels (Darmi and Albion, 2013)

Darmi, R., & Albion, P. (2013). English language in the Malaysian education system: Its existence and implications. In Proceedings of the 3rd Malaysian Postgraduate Conference (MPC 2013) (pp. 175-183). Education Malaysia.

Comment:
6. Were only some of the items selected from the original questionnaires? Any implications on validity and reliability?

Response:

Yes, some of the items were selected/adapted from the original questionnaires to suit this study. Appendix A shows the measure for the dependent variable, i.e. RACB, which consists of 3 items adapted from Ajzen (1991) and Murugesan (2008). Appendix B shows the measures for all TPB factors, which consist of 15 items adapted from Ajzen (1991), Boldero (1995) and Tonglet et al. (2004), and Murugesan (2008). Appendix C shows the measures for all VBN factors, which consist of 33 items adapted from Stern et al. (1999) and Steg et at. (2005). Appendix D shows the measures of habit factor, which consists of 3 items adapted from Venkatesh et al., (2012) and Triandis (1980). All items are measured using Likert 5-point scale, i.e. strongly disagree (1), disagree (2), neutral (3), agree (4) and strongly agree (5). The items were adopted from past studies. Some wording was changed to suit the present context. The validity and reliability of the constructs were tested using confirmatory factors analysis (CFA) and Cronbach’s alpha. In the final model, the composite reliability (CR) for all factors is above 0.70 which shows acceptable internal consistency (Nunnally & Bernstein, 1994). The Average Variance Extracted (AVE) for all factors is above 0.5 which shows acceptable convergent validity. Table 3 shows the √AVE values (see the diagonal values) of each construct is greater than the correlation values in the same row. This shows high discriminant validity. There were no multicollinearity issue in the model since all the correlations are below 0.8 (Sekaran, 2000). Cronbach Alpha (i.e. a reliability test statistic) was calculated for all factors and they were above the threshold value of 0.7 (Sekaran, 2000). Thus, there was no implication on the validity and reliability.

Comment:
7. Please also include validity and reliability scores of the original questionnaires.

Response:

The following explanation is added:

Prior to the pilot study, the original questionnaires were pretested for content validation by 10 experts who are researchers, lecturers and professor. Based on their feedback, amendments were made on the questionnaire content, format, terms, ease of completion and comprehensibility. A pilot study was then conducted with 100 questionnaires distributed in public places. Cronbach Alpha (i.e. a reliability test statistic) was calculated for all factors and they were above 0.7.

Comment:
8. “As this study focused only on the main effects of the TPB and VBN variables, future studies could include demographic variables such as age, gender or income, which can be useful in providing meaningful information.”
Please clarify why such basic demographics were not collected.

Response:

In fact, the basic demographic variables were collected in this study. However, this study focused only on the main effects of the TPB and VBN variables. Due to the limited space, we plan in the future studies to analyze the impacts of demographic variables such as age, gender or income, as moderating variables on main relationships, which may be useful in providing meaningful information for manufacturers, marketers and policy makers to facilitate them to promote high-cost green computers among consumers from different groups.
