# Supplementary material for: Antecedents of the responsible acquisition of computers behaviour: Integrating the theory of planned behaviour with the value-belief-norm theory and the habits variable
Source: PLoS One. 2023 Jun 2;18(6):e0286022. doi: 10.1371/journal.pone.0286022 (PMC10237383; doi:10.1371/journal.pone.0286022)
Supplement: Attachment — Submitted filename: Response to Reviewer Comments.docx [file pone.0286022.s005.docx]

**Antecedents of the responsible acquisition of computers behaviour: Integrating the theory of planned behaviour with the value-belief-norm theory and the habits variable**

**Response to Comments**

**Reviewer #3:**

**Comment**

About model and originality of the paper: The authors select “RACB” as research subject on the topic of green computer purchase behavior in Malaysia. However, the authors don’t explain why these customers should be considered individually and don’t introduce any new indicators in this research filed. Also, the research model in the study is so complicated which not suitable for the SEM model. it may be necessary for the author to simplify the model or to consider using a different type of statistical analysis.

**Response:**

Thank you for the comments.

i. We included the following justifications for choosing individual consumers as the sample in Section 3.4.

“Individual consumers were chosen as the unit of analysis because 40% of environmental degradation is attributed to individual consumers’ irresponsible consumption of non-sustainable products (Joshi and Rahman, 2017). ”

ii. Why new indicators/constructs are not introduced?

As it is, there are already sufficient 15 constructs from the Theory of Planned Behaviour, Value Belief Norms theory and habit construct. There is no need of adding new construct. There is no need of developing new variables as the existing theories (TPB and VBN) can explain RACB.

iii. Regarding the appropriateness of SEM technique, we added the below justifications as to why SEM technique is adopted in Section 3.6. Therefore, we didn’t change the model or use other statistical analysis technique.

“SEM tests a large number of independent and dependent constructs statistically in a simultaneous analysis to develop a structural model, which is a plausible representation of the relations between all variables (Hair et al., 2014). SEM is a comprehensive approach of data analysis whereas traditional multivariate procedures are incapable of either assessing and correcting measurement error. SEM provides explicit estimates of the measurement error through confirmatory hypothesis-testing approach (Hair et al., 2014). Many researchers like Gao and Bai (2014); Kiatkawsin and Han (2017); Vincent et al. (2022); Nguyen (2022) etc. validated the causality among the independent and dependent constructs in the integrative research framework via SEM technique.”

- Hair, J. F., Jr., Black, W. C., Babin, B. J., & Anderson, R. E. (2014). Multivariate data analysis (7th ed.). Essex: Pearson Education.
- Gao, L., Bai, X. (2014). A unified perspective on the factors influencing consumer acceptance of internet of things technology. Asia Pacific Journal of Marketing and Logistics, 26(2), 211-231.
- Kiatkawsin, K., & Han, H. (2017). Young travelers' intention to behave pro-environmentally: Merging the value-belief-norm theory and the expectancy theory. Tourism Management, 59, 76-88.
- Vincent, K.E., Shuliang, Z., Benjamin, K. L (2022). Investigating household waste separaction behaviour: the salience of an integrated norm activation model and the theory of planned behaviour, Journal of Environment Planning and Management.
- Nguyen, T. P. L. (2022). Intention and behaviour toward bringing your own shopping bags in Vietnam: integrating theory of planned behaviour and norm activation model”, Journal of Social Marketing, 12(4), 395-419.

iv. it may be necessary for the author to simplify the model

In fact, in the final revised model of SEM as shown in Fig. 2, the model is simplified from the original 15 constructs to 9 constructs as 6 constructs do not have any significant relationships., i.e. Attitude Towards Responsible Acquisition of Computers, Perceived Behavioural Control, Egoistic, Altruistic, Awareness of Consequences – Egoistic and Awareness of Consequences – Altruistic.

**Comment:**

Literature review: The literature part is superficial and shallow even in the revised version. For example, the paper does not provide much detail or depth on the topic that what the weaknesses associated with the use of a single theory by the other authors one by one and the contributions to integrate TPB variables with VBN variables to explain RCB. Moreover, some updated and related literature about TPB & responsible (purchase) behavior were not mentioned/cited yet. I suggest the author(s) to consider reading and citing the following impactful, and updated literature; sure the authors can cite other similar papers not limit to listed ones.

• Gao, L., Bai, X. (2014). A unified perspective on the factors influencing consumer acceptance of internet of things technology. Asia Pacific Journal of Marketing and Logistics, 26(2), 211-231.

• Liu, M., Liu, Y., Mo, Z. (2020). Moral norm Is the key: An extension of the theory of planned behaviour (TPB) on Chinese consumers’ green purchase intentions, Asia Pacific Journal of Marketing and Logistics, 32(8), 1823-1841.

• Kumagai, K. (2021). Sustainable plastic clothing and brand luxury: a discussion of contradictory consumer behaviour, Asia Pacific Journal of Marketing and Logistics, 33(4), 994-1013

• Liu, M., Liu, Y., Mo, Z., Zhao, Z., Zhu, Z. (2020). How CSR influences customer behavioural loyalty in the Chinese hotel industry, Asia Pacific Journal of Marketing and Logistics, 32(1), 1-22.

• Wang, Y., Ko, E., & Wang, H. (2022). Augmented reality (AR) app use in the beauty product industry and consumer purchase intention. Asia Pacific Journal of Marketing and Logistics, 34(1), 110-131.

• Mo. Z., Liu M., Liu, Y. (2018). Effects of functional green advertising on self and others, Psychology & Marketing. 35(5), 368-382.

**Response:**

Thanks for the comments.

We have added the following new paragraphs to enhance the richness of the literature review regarding the TPB theory in terms of its applications, strengths and weakness along with the shortcoming of relying on single theory. We also have updated the literature review with the latest published articles, including the suggested articles. Please refer to the introduction (Section 1):

“Many studies have been conducted to discover the antecedents of RCB using various theories and factors such as the theory of planned behaviour (TPB), the value-belief-norm (VBN) theory, and the habitual factor (Gao& Bai, 2014; Liu, Liu & Mao, 2020; Kumagai, 2021; Mo, Liu & Liu, 2018). The TPB articulates how individuals make reasoned choices and choose alternatives with the greatest benefits against the lowest costs in serving their self-interest, wherein the behaviour is determined by behavioural intention that lies on individual positive assessment on the behaviour (attitude), social pressure adopting the behaviour (subjective norm) and perceived ease of engaging the behaviour (perceived behavioural control) (Kumagai, 2021).

Prior studies substantiated that the TPB could serve as a basic model in explaining RCB encapsulating energy saving (Gao & Bai, 2014; Gao et al., 2017; Liu, Liu & Mao, 2020; Ru et al., 2018), recycling (Wan et al., 2017), purchasing organic food (Yazdanpanah and Fourouzani, 2015), green purchase (Kumagai, 2021; Mo, Liu & Liu, 2018), etc. However, the TPB owns its limitation i.e., TPB is a self-interest theory that primarily comprises rational predictors while omitting other relevant RCB contextual factors like moral obligation, habits, etc. (Yurie et al., 2020); thus, affecting the predictive power of the theory. This is evidenced in Yazdanpanah and Fourouzani (2015)’s findings, where the predictive power of the TPB became higher from 56% to 65% after adding moral norms and self-identity to the original TPB. Additionally, Gao et al. (2017)’s studies revealed that the explanatory power of TPB raised from 22.6% to 34.9%, and the significant predictors of individual energy-saving behaviour include all the TPB variables (except subjective norm) and the added variables i.e., descriptive norms and personal norms. Wan et al. (2017)’s studies found two additional variables, i.e., awareness of consequences and moral norms, increased the predictive power of the TPB in predicting recycling intention. Liu et al. (2019) revealed the extended TPB model performed well in green purchase intention setting. All these findings shed light for future RCB research that extending TPB is required as the theory fails to consider the moral aspect; thus, it cannot fully explain the behaviour.

RCB is a pro-social behaviour which is not merely predicted by TPB variables, but also could be well-explained by VBN theory, wherein the VBN theory proposes that altruistic behaviour is triggered by the morality factors such as personal norms, ascriptions of responsibility, awareness of consequences, new ecological paradigm, and values. However, VBN theory does not cover the self-interest predictors that are captured by TPB variables. So far, most RCB researches focus mainly on extending TPB with one or a few variables instead of integrating the whole TPB with the complete VBN theory to provide a holistic view of how the variables of both theories work, notably in high-cost RCB context (e.g. the RACB of present study).The findings of Ates (2020) revealed that integrating TPB variables with morality variables accounts for more explained variance in pro-environmental behaviour (R^2^=0.488) compared to the use of individual TPB (R^2^=0.464). Ates (2020) highlighted that other important variables (e.g., egoistic value, altruistic value, new ecological paradigm belief) presented in the VBN should be taken into consideration. However, Ates’ (2020) study is confined to general RCB, not specific behaviour like green purchase behaviour. Another study by Li et al. (2021) combined the TPB, the diffusion of innovation theory (DOI), and the personal norm variable into a conceptual framework. However, the integration of the whole VBN variables were excluded from their study.

Although the previous literature highlighted that habits should be included in studies for a more accurate prediction of repetitive behaviour, this factor had been ignored in the TPB and VBN models (Jansson et al., 2010; Verplanken & Aarts, 1999; Cheung et al., 1999). In response, extensive research added habits into the TPB model. For instance, Cheung et al. (1999) revealed that the explained variances of the TPB increased from 32.5% to 67.4% after incorporating habits into the TPB model to explain wastepaper recycling behaviour. Besides, the impact of TPB variable i.e. SN on VBN factors (e.g. AR, and PN), and habits are under-explored (Khan et al., 2022).

Moreover, most of the prior RCB studies that combined the TPB and the VBN theory were focused on general pro-environmental behaviour (Ates, 2020; Bamberg & Moser, 2007), or low-cost, pro-environmental behaviour in the choice of transportation (Heath & Gifford, 2002; Bamberg & Schmidt, 2003) and the purchase of organic products (Li et al., 2021). The present study considers high-cost pro-environmental behaviour in the purchase of green computers that are more expensive than conventional computers.

To address the above research gaps, the objective of the study is to investigate the antecedents of RACB by integrating the TPB and the VBN theory with the habits variable. The outcome of this research will provide new knowledge of how the theories can be integrated to explain RACB. With this understanding, recommendations can be provided to policymakers, NGOs, manufacturers, and marketers to promote sustainable consumption through RACB.”

**Comment:**

Methodology and result presentation: The process of data analysis in this paper is too simple and rough. The author needs to present each step of data analysis in the concise way. Also, the title of table 4 need to be revised. The author didn’t present the direct and indirect effects clearly. Please enhance the mentioned parts.

**Response:**

Thank you for your feedback.

We are following the two-stages approach proposed by Anderson and Gerbing (1988) for SEM analysis. We added the following in Section 3.6:

“As suggested by Anderson and Gerbing (1988), the two-step approach was used in this study. In the first step, the measurement model was analysed for adequacy, and confirmatory factor analysis (CFA) was used to test the validity and reliability of the measurement model. The second step involved testing the structural model and hypotheses through assessing the path coefficients for each hypothesised relationship.”

The title of table 4 has been revised. We only test the direct effect.

**Comment:**

The integration of the TPB and VBN models cannot be seen as the theoretical contribution. Without having enough implications/contributions associating with your core concepts (TPB or VBN), the value of the work will be much weakened. Please enhance your implications/contributions parts in resubmission.

**Response:**

Thank you for your feedback.

The theoretical contribution is not just the integration of the TPB and VBN. In this study, we included the **complete** model for both the TPB and VBN theory and added the habit variable and the research was conducted in the context of high-cost purchase decisions e.g. acquisition of individual computers in emerging economy (i.e. Malaysia). **This study unveils the complex relationships among the two models and the habit variable. Thus, this contribution is unique**.

The following paragraphs have been rewritten to strengthen the theoretical implications (section 5.5) of this study:

“This study enriches the literature about responsible purchasing behaviour by addressing the absence of empirical studies examining the level of RCB and the antecedents of adopting RCB in the context of high-cost purchase decisions related to the acquisition of computers by individual consumers, particularly in emerging economy (i.e. Malaysia) comprised of low- and middle-income groups, fast-growing computer penetration (Worldometers, n.d.), and collectivist cultures.

This study provides holistic views relating to the integration of the complete TPB and VBN models with the habits variable in the high-cost pro-environmental behaviour domain, filling the literature gap where most of the scholars either dived into individual models like TPB (e.g., Lago, 2020; Kang & Moreno, 2020) or VBN (e.g., Sharma and Gupta, 2020), or extended individual models with additional variables (e.g., Ates, 2021). It unveils the complex relationships among the two models and the habits variable. Thus, this contribution is unique.

The present findings confirm that a cognitive deliberative process occurs and that self-interest variables are significant when environmentally responsible behaviour is costly. Also, biospheric values are the main trigger of the TPB’s self-interest variable and VBN’s morality variable in forming decisions to adopt RACB. This further re-affirms that biospheric values are distinct from altruistic values in the context of pro-environmental behaviour (Nguyen, 2016; Ates, 2021). SN are a direct predictor of RACB (TPB variable), PN (VBN variable), AR (VBN variable), and HA (TIB). This further substantiates that SN are a particularly important element in the context of developing countries with collectivist cultures. In addition, HA affects intention but not RACB directly. This implies that individuals’ decisions will be affected by their HA in reasoning and assessing the costs and benefits of each alternative (i.e., RACBI) in the context of high-cost pro-environmental behaviour.”

**Comment:**

There is a lot of inappropriate/non-typical English/nonsense expressions, the language is not academic-oriented enough. Some tables in the paper should be formatted consistently with three-line format. Before re-submission, I strongly suggest the author need to send the draft to proofreader/copy-editor for improvement.

**Response:**

Thanks for the comments. We corrected the table and sent the manuscripts for proofreading.
